# Salvianolic Acid B Attenuates Apoptosis of HUVEC Cells Treated with High Glucose or High Fat via Sirt1 Activation

**DOI:** 10.1155/2019/9846325

**Published:** 2019-04-21

**Authors:** Jinghui Zhai, Lina Tao, Yueming Zhang, Huan Gao, Xiaoyu Qu, Yanqing Song, Sixi Zhang

**Affiliations:** Department of Pharmacy, The First Hospital of Jilin University, Changchun 130021, China

## Abstract

High glucose and high fat are important inducements for the development and progression of diabetic cardiopathy. Salvianolic acid B (SAB), which is the most abundant and bioactive compound in Danshen, attenuates oxidative stress-related disorders, such as cardiovascular diseases, cerebral ischemia, and diabetes. However, the effect of SAB on diabetic cardiopathy is not clear. The aim of study was to investigate the effect and the underlying molecular mechanisms of SAB on diabetic cardiopathy in vitro model. The human umbilical vein endothelial (HUVEC) cells were treated with high glucose (HG, 30 mM) or high fat (palmitic acid, PA, 0.75 mM) in the presence or absence of SAB (100, 200, and 400 mg/L) and incubated for 24 h. We found that HG or PA induced apoptosis of HUVEC cells, while treatment with SAB inhibited the apoptosis. We also found that SAB reversed HG- or PA-induced oxidative stress, apoptosis cell cytokines production, and expression of thioredoxin-interacting protein (TXNIP). Moreover, SAB increased HG- or PA-induced expression of Sirtuin 1 (Sirt1), a nicotinamide adenine dinucleotide- (NAD^+^-) dependent histone deacetylase. Exposure of HUVEC cells to Ex527 (Sirt1 inhibitor) suppressed the effect of SAB on acetyl-p53 and procaspase-3 expressions. In conclusion, the results suggested that SAB could attenuate HUVEC cells damage treated with HG or PA via Sirt1 and might be a potential therapy agent for the diabetic cardiopathy treatment.

## 1. Introduction

Diabetes mellitus (DM) is a metabolic disease with high worldwide incidence (4-5%). DM patients compared with nondiabetic people bear up to sixfold higher risk of cardiovascular disease [1]. Endothelial dysfunction induced by glucotoxicity and lipotoxicity, which is a common problem in DM, has an important role in cardiovascular diseases [2]. Endothelial dysfunction results in increased oxidative stress and elevated levels of inflammatory markers due to increased oxygen free radical generation, lipid peroxides formation, impaired glutathione metabolism, and impaired antioxidant defense systems [3, 4]. Thus, endothelial dysfunction is the early feature of cardiovascular complications in DM.

**Figure 1:**
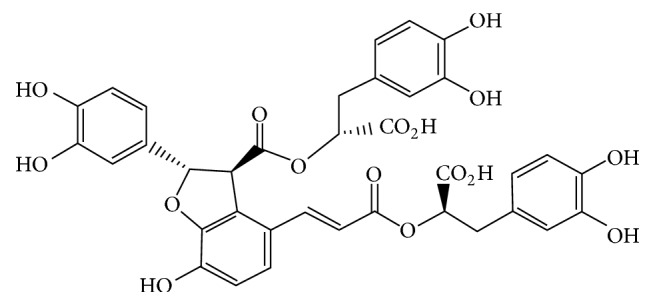
Chemical structure of salvianolic acid B (SAB). Molecular formula: C36H30O16. Molecular weight: 718.6138.

The dried root of* Salvia miltiorrhiza *Bunge (also known as Danshen) is popular in traditional Chinese medicine and has been used extensively to treat various diseases, including cerebrovascular diseases, coronary artery diseases, and myocardial infarction [5–8]. Salvianolic acid B (SAB; molecular formula: C36H30O16; Figure 1) is the most abundant and bioactive compound in Danshen and appears to have antioxidative and neuroprotective activities in vivo and in vitro [9–12]. Multiple studies have shown that SAB is used as a potent reactive oxygen species (ROS) scavenger and effectively attenuates oxidative stress-related disorders, such as cardiovascular diseases, cerebral ischemia, and diabetes [13–15]. It has been demonstrated that oxidative stress could accelerate the progress of diabetic cardiopathy. Excessive oxidative stress activates multiple intracellular signaling pathways and stimulates transcription factors, thus resulting in apoptosis [16]. Therefore, it is necessary to explore antioxidative agents for the diabetic cardiopathy treatment. Thioredoxin-interacting protein (TXNIP) has an important role in cellular metabolism. Increasing researches suggested that relationship of TXNIP and DM is close [17, 18]. It is an initial stage highly induced by diabetes and hyperglycemia. Furthermore, an important function of TXNIP is to interact with thioredoxin (TRX) and reduce ROS [19, 20].

Previous studies confirmed that SAB protected human endothelial cells from oxidative stress-induced cellular damage [21]. SAB is a potent activator of Sirt1 in a variety of disease models [13, 22, 23]. Sirtuin 1 (Sirt1), a nicotinamide adenine dinucleotide- (NAD+-) dependent histone deacetylase, is involved in the regulation of metabolism, cell survival, differentiation, and longevity and exerts beneficial effects on glucose-lipid homeostasis and insulin secretion in diabetic patients [24]. Thus, it has been suggested that Sirt1 is a key regulator of vascular senescence and dysfunction. It has been reported that the activation of Sirt1 protects against vascular dysfunction in mice with diabetes [25]. However, it is not clear whether SAB can protect human umbilical vein endothelial (HUVEC) cells from high glucose or high fat associated with activating Sirt1.

Therefore, we hypothesized that SAB might benefit HUVEC cells against high glucose or high fat via Sirt1 activation. To address the hypotheses, this study was aimed to investigate the potential effects of SAB on high glucose or high fat-induced HUVEC dysfunction. Firstly, we verified that SAB promoted HUVEC cells proliferation by MTT assay and inhibited apoptosis by flow cytometry assay, mitochondrial membrane potential assay, and western blotting assay. Secondly, SAB protected HUVEC cells from oxidative stress though suppressing expression of TXNIP. Finally, we found that Sirt1 had an important role in SAB attenuating apoptosis of HG- or PA-induced HUVECs cells by present or absent Ex527 (Sirt1 inhibitor).

## 2. Materials and Methods

### 2.1. Cell Culture

HUVECs were incubated in endothelial cell medium (ECM, Sciencell) containing endothelial cell growth supplement (ECGS), 10 % fetal bovine serum (Sciencell), 100 U/L penicillin, and 100 mg/ml streptomycin (Sciencell) and incubated at 37°C in 5 % CO_2_. Cell cultures were split once every 2 days.

### 2.2. Cell Proliferation Detection Assay

HUVECs were seeded in 96-well plate with the density of 5×10^4^ cell/well. Cells were treated with normal glucose (5.5 mM glucose) or 10 mM glucose and 20 mM glucose or high glucose (30 mM glucose) in the presence or absence of SAB (100 mg/L, 200 mg/L, or 400 mg/L) (≥98%, Zhongshanlianjiu Biotechnology, Zhongshan, China). The other cells were treated with palmitic acid (PA, Sigma) (0.25, 0.5, and 0.75 mM) or 0.75 mM PA in the presence or absence of SAB (100 mg/L, 200 mg/L, or 400 mg/L). After 4h, 12h, or 24h, 10 *μ*L of 5 mg/mL MTT (3-[4, 5-dimethylthiazol- 2-yl]-2, 5-diphenyltetrazolium bromide, Sigma) solution was added per well and incubated for 4h. Subsequently, the supernatant was removed and 150 *μ*L DMSO was added to dissolve the formed formazan. The absorbance value was detected at 490 nm using a microplate spectrophotometer (ELX800).

### 2.3. Flow Cytometric Analysis of Cell Apoptosis

HUVECs were plated in 6-well plate overnight and treated with high glucose or PA in the presence or absence of SAB for 24 h. At the end of treatment, the cells were collected and stained with Annexin V-FITC/PI apoptosis detection kit (Beyotime Biotechnology) according to manufacturer's instructions [26].

### 2.4. Mitochondrial Membrane Potential Assay

HUVECs were plated in 6-well plate overnight and treated with high glucose or PA in the presence or absence of SAB for 24 h. JC-1 easily penetrates cells and healthy mitochondria. Briefly, after treatment, the cells were incubated at 37°C for 1 h with 5 mg/L JC-1 (Beyotime Biotechnology), then washed twice with PBS, and placed in fresh medium without serum. Lastly, images were captured with a fluorescence microscope (Olympus BX83, Olympus, Japan). The ratios of red/green fluorescent densities were calculated.

### 2.5. Detection of Oxidative Stress

The reactive oxygen species (ROS) level was evaluated using a Reactive Oxygen Species Assay Kit of DCFH-DA (Jiangcheng Bioengineering Institute, Nanjing, China) according to the manufacturer's instruction. The fluorescence intensity was analyzed with a microplate reader (Tecan, Switzerland) at an excitation wavelength of 488 nm and at an emission wavelength of 525 nm.

### 2.6. Western Blotting

HUVECs were seeded into Petri dishes (50 mm×50 mm) and treated with high glucose (30 mM) or PA (0.75 mM) in the presence or absence of SAB (100 mg/L, 200 mg/L, or 400 mg/L) for 24 h. Then, the cells were extracted using lysis buffer (1 mM PMSF, 50 mM Tris, 1%SDS, sodium pyrophosphate, *β*-glycerophosphate, sodium orthovanadate, sodium fluoride, EDTA, leupeptin, and other inhibitors) (Beyotime Biotechnology, Shanghai, China). The protein concentration was detected using BCA assay kit (Beyotime Biotechnology). The lysates were then denatured at 100°C for 5 min. 20 *μ*g proteins of each group were separated on SDS-PAGE, and then transferred onto PVDF membranes (Bio-Rad, CA). Membranes were blocked with 5% nonfat milk for 1 h at room temperature and probed with primary antibodies against Bcl-2 (1:2000, Cell Signaling Technology), caspase-3 (1:2000, Cell Signaling Technology), caspase-9 (1:2000, Cell Signaling Technology), TXNIP (1:2000, Santa Cruz Biotechnology, Inc.), acetyl-p53 (1:2000, Abcam) and Sirt1 (1:2000, Abcam), and GAPDH (1:5000, Hangzhou Goodhere Biotechnology Co., Ltd.) at 4°C overnight. Then the membranes were washed with TBST and incubated with HRP-conjugated secondary antibodies (1:5000, Beyotime Biotechnology) for 1 h at room temperature. Finally, the blots were imaged with ECL (Amersham, UK) and visualized on X-ray film (Kodak, China). The scanned digital images were quantified using ImageJ 1.37c software. All bands were normalized to GAPDH levels.

### 2.7. Immunofluorescence

Indirect immunofluorescence staining was used to observe the expression and localization of proteins, including Sirt1 (1:200) and TXNIP (1:200), in HUVECs. HUVECs were plated in 12-well plate overnight, and treated with SAB in combination with high glucose or PA for 24 h. After washing 3 times with cold PBS, HUVECs were fixed in 4% paraformaldehyde for 10 minutes at room temperature and then washed 3 times with PBS. After treatment with Triton X-100 and blocking with 5% BSA, HUVECs were then incubated with primary antibodies overnight at 4°C. After washing with PBS, HUVECs were incubated with corresponding FITC coupled secondary antibodies (Absin, Shanghai, China) for 1 h and counterstained with DAPI (Beyotime Biotechnology) for 10 min. Images were captured with a fluorescence microscope (Olympus BX83, Olympus, Japan).

### 2.8. Statistical Analysis

GraphPad Prism 5 software was used for all statistical analysis. Data are expressed as means ± standard error of the mean (SEM) of three or more independent experiments. The unpaired Student's t-test or one-way ANOVA followed by Tukey test was used for pairwise comparisons among groups where appropriate, with significance established as p < 0.05. 

## 3. Results

### 3.1. SAB Inhibited HG- or PA-Induced Proliferation and Apoptosis of HUVEC Cells

**Figure 2:**
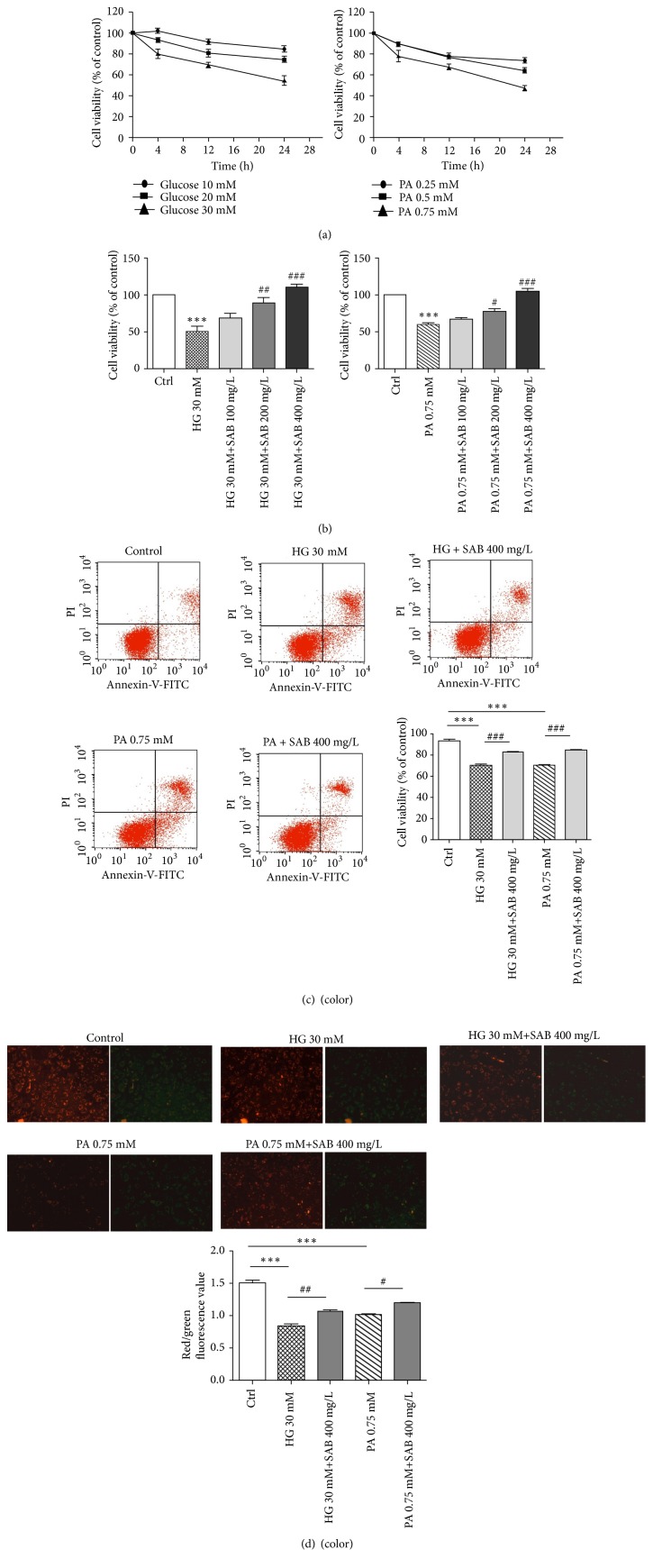
SAB reverses the inhibitory effects of high glucose or high fat (PA) on HUVEC cells proliferation and apoptosis. (a) Effect of glucose (10, 20, and 30 mM) and PA (0.25, 0.5, and 0.75 mM) on the percentage of survival in HUVECs at 4, 12, and 24 hours by MTT assay; (b) effect of HG (30 mM) or PA (0.75 mM) with SAB (100, 200, and 400 mg/L) on cells viabilities; *∗∗∗p* <0.001 versus control group, ^#^*p *<0.05 versus HG or PA group,^ ##^* p *<0.01 versus HG or PA group, and^ ###^* p *<0.001 versus HG or PA group. (c) Effect of SAB on apoptosis induced by HG or PA in HUVECs after Annexin V and PI double staining; *∗∗∗p* <0.001; ^###^* p *<0.001. (d) JC-1 staining assay for mitochondria membrane potential. SAB remained mitochondria membrane potential in HG- or PA-induced HUVEC cells; *∗∗∗p* <0.001; ^#^*p *<0.05, ^##^* p *<0.01.

HUVEC cells were cultured under the conditions of glucose (10, 20, and 30 mM) or palmitic acid (PA, 0.25, 0.5, and 0.75 mM) for 4 h, 12 h, and 24 h. Cells proliferation was evaluated using the MTT assay. The results in Figure 2(a) suggested that both high glucose and high fat-induced cell viability decrease by concentration gradient and time gradient. The doses of 30 mM glucose and 0.75 mM PA treated for 24 h were used for further experiments. We found a dose dependent increase in cell viability upregulating exposure to coadministration of SAB (100, 200, and 400 mg/L) and HG or PA (Figure 2(b)). From Figure 2(c), HUVEC cells apoptosis induced by HG or PA were prevented by SAB significant (P<0.001).

Mitochondria, as the primary site of cellular energy generation and oxygen consumption, represent a likely pathway for HG- or PA-induced apoptosis [27]. In this study, we observed that SAB significantly prevented impaired mitochondrial bioenergetics by retaining mitochondria membrane potential (Figure 2(d)).

### 3.2. SAB Reverses the Effects of HG or PA on HUVEC Cells ROS Production

**Figure 3:**
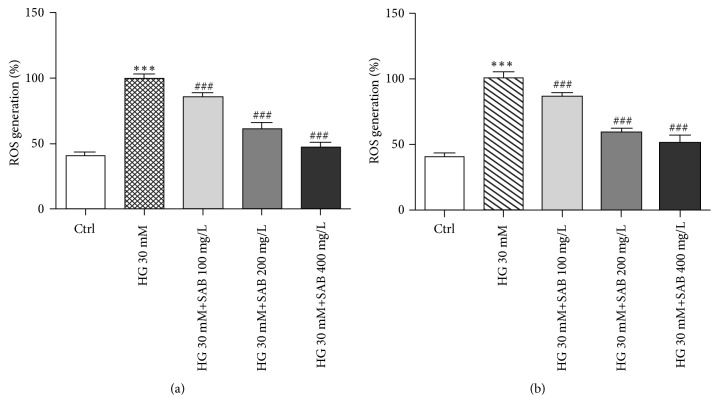
SAB reverses the effects of HG or PA on HUVEC cells ROS production. (a) Effect of SAB (100, 200, and 400 mg/L) with HG on ROS generation in HUVEC cells. (b) Effect of SAB (100, 200, and 400 mg/L) with PA on ROS generation in HUVEC cells. *∗∗∗ p* <0.001 versus control group, ^#^* p* <0.05 versus HG or PA group, and ^###^* p* <0.001 versus HG or PA group.

As oxidative stress is crucial for the HG- or PA-induced cell injury, level of ROS were assessed. As shown in Figure 3, compared to the control group, intracellular ROS levels in HUVEC cells were markedly elevated in response to HG or PA treatment, and the increase was inhibited by treatment with SAB.

### 3.3. SAB Reduces Apoptosis Related Proteins and Involves Bcl-2, Procaspase-3, and Procaspase-9 Activation

**Figure 4:**
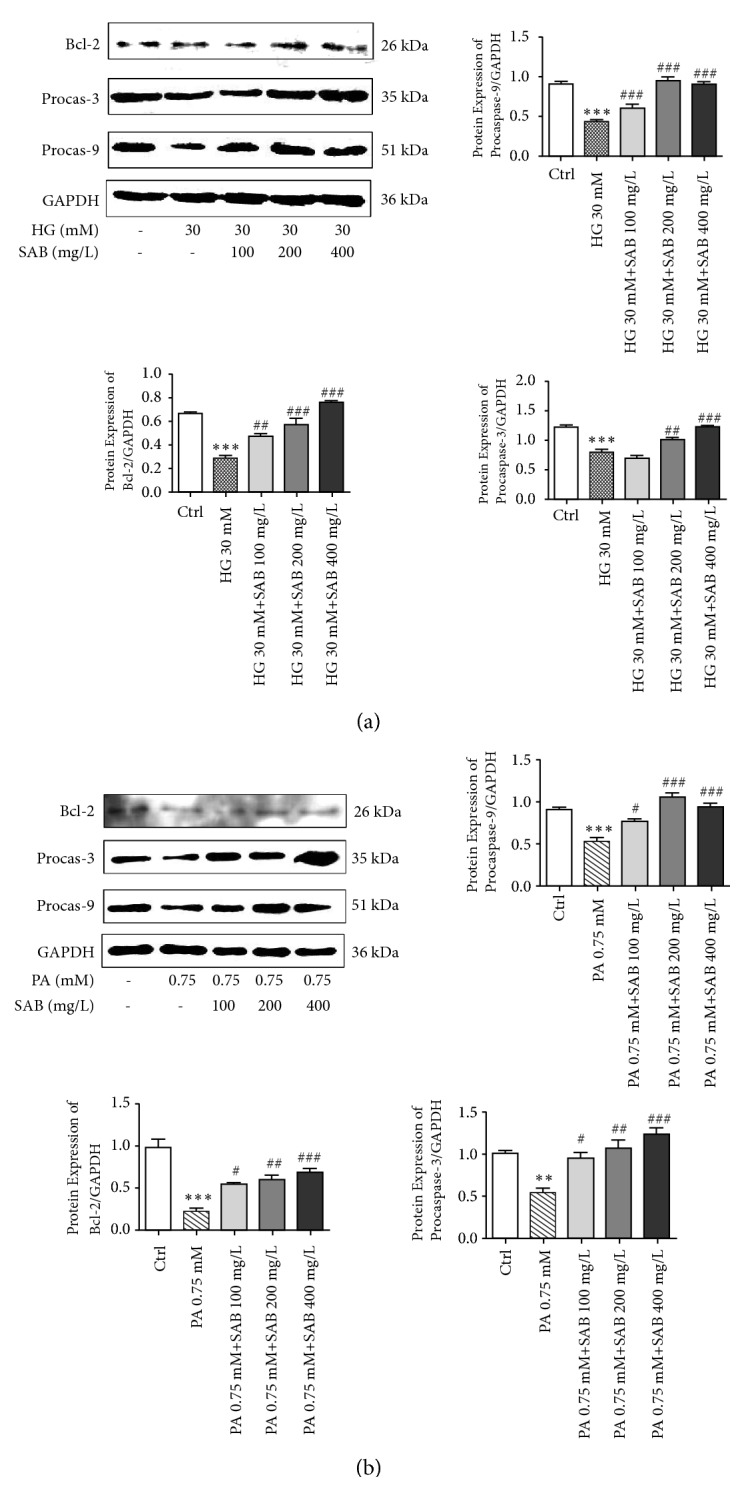
SAB increased expressions of procaspase-3, procaspase-9, and Bcl-2 in response to HG (a) or PA (b) conditions. *∗∗∗ p* <0.001 versus control group, ^#^* p* <0.05 versus high glucose group,^ ##^* p* <0.01 versus high glucose group, and ^###^* p* <0.001 versus high glucose group.

To determine whether the protective effects of SAB are associated with apoptosis, we measured the levels of Bcl-2, procaspase-3 and procaspase-9 expressions. HG or PA conditions decreased expression of Bcl-2, procaspase-3, and procaspase-9. However, all these effects were reversed dose-dependently by SAB (Figures 4(a) and 4(b)). These results indicate that SAB inhibits apoptosis induced by HG or PA associated with Bcl-2, caspase-3, and caspase-9 proteins.

### 3.4. SAB Modulates the Expression of TXNIP and Sirt1

**Figure 5:**
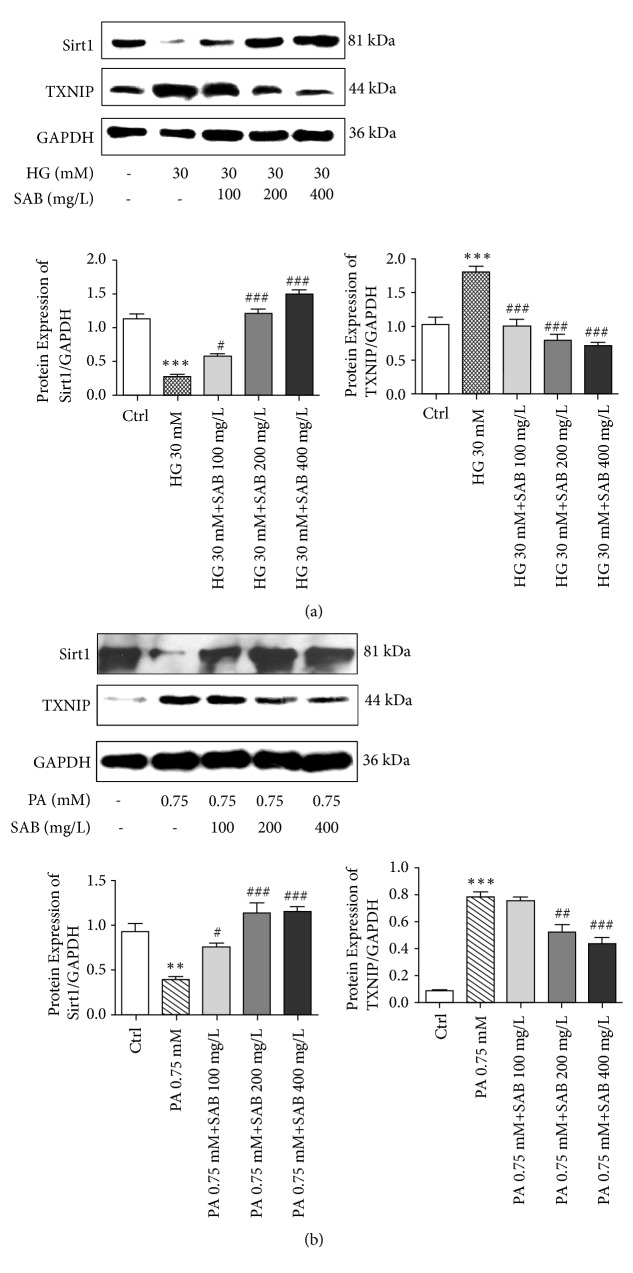
SAB modulates the expression of Sirt1 and TXNIP in HUVEC cells treated by HG (a) or PA (b). *∗∗∗ p* <0.001 versus control group, *∗∗ p* <0.01 versus control group, ^#^* p* <0.05 versus HG or PA group, ^##^* p* <0.01 versus HG or PA group, and ^###^* p* <0.001 versus HG or PA group.

**Figure 6:**
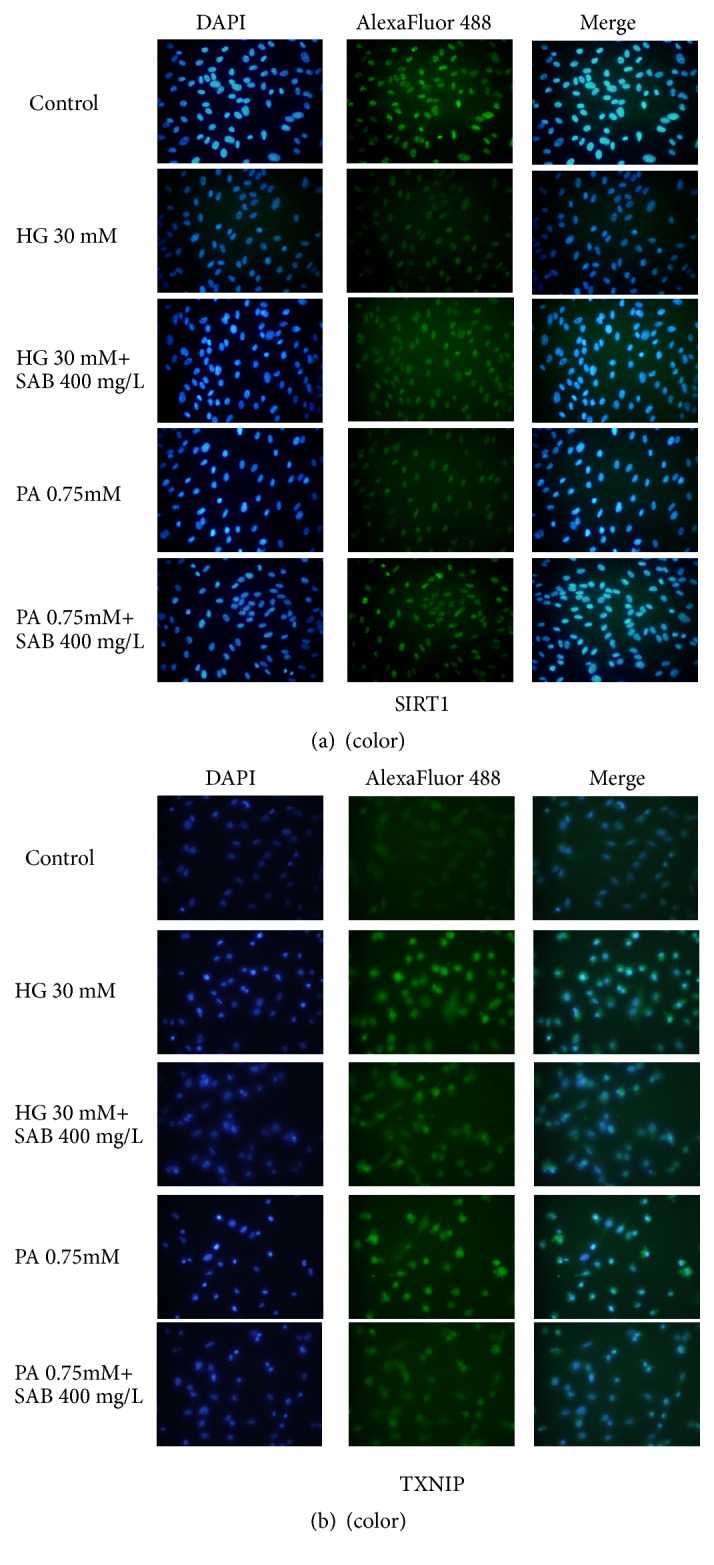
Effect of SAB (400 mg/L) on fluorescence intensity of Sirt1 (a) and TXNIP (b) in HG- or PA-induced HUVEC cells.

To explore the related mechanism of the SAB effect in HG- or PA-induced HUVEC cells, the expression levels of TXNIP and Sirt1 were detected by western blotting. TXNIP, which is the thioredoxin binding protein, acts as a mediator of cellular metabolism. It was found that TXNIP mediates glucose-induced apoptotic death in pancreatic beta cells [28, 29]. As shown in Figure 5, we found that HG or PA conditions reduced the expression levels of Sirt1 (*p* < 0.001;* p* < 0.005), an effect that was reversed by SAB treatment (*p* < 0.001;* p* < 0.01). Additionally, compared to the control group, TXNIP expression was significantly increased in response to HG or PA stimulation (*p* < 0.001) but was decreased in SAB-treated cells (*p* < 0.001;* p* < 0.005). Fluorescence intensities of Sirt1 and TXNIP coincided with results of western blotting (Figure 6).

### 3.5. Inhibition of Sirt1 Expression Reversed the Effect of HG or PA and SAB on HUVEC Cells

**Figure 7:**
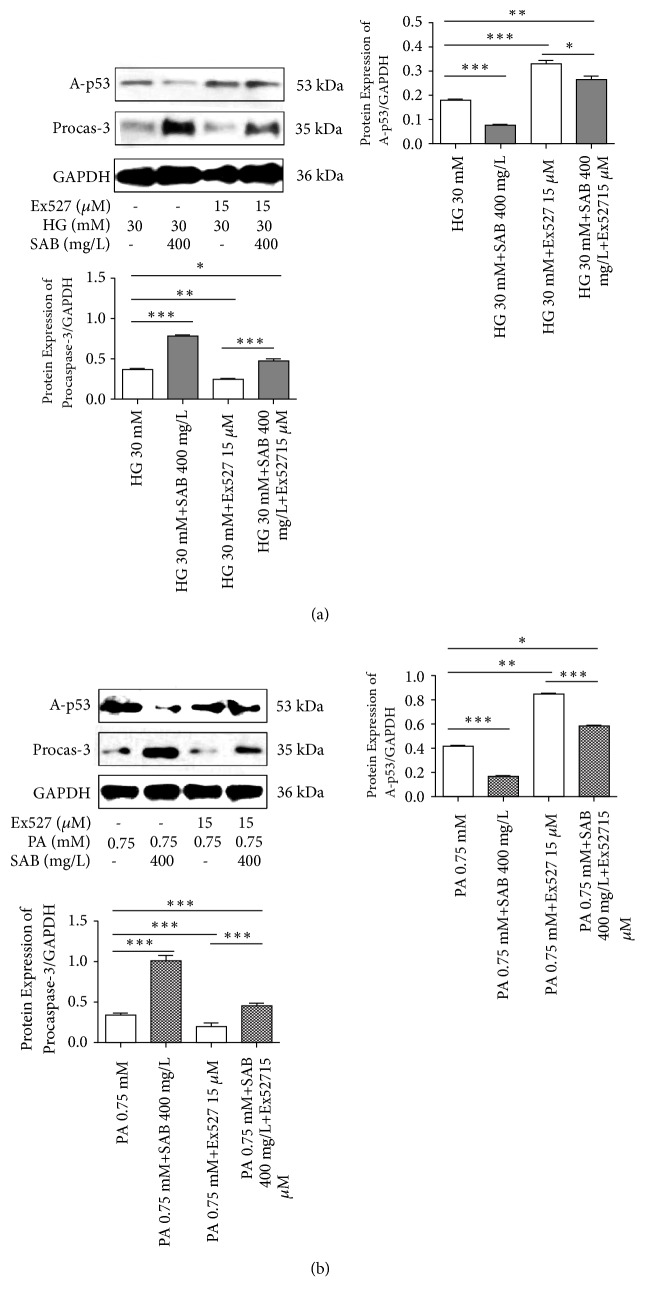
Effect of SAB was reversed on expressions of acetyl-p53 and procaspase-3 by Ex527 (15*μ*M) in HG (a)- or PA (b)-induced HUVEC cells. *∗ p* <0.05 versus control group, *∗∗ p* <0.01 versus control group, *∗∗∗ p* <0.001 versus control group, ^#^* p* <0.05 versus Ex527 group, and ^##^* p* <0.01 versus Ex527 group.

To evaluate the relation among Sirt1 in the effect of SAB in HG- or PA-induced HUVEC cells, the HUVEC cells were incubated with the Ex527 (inhibitor of Sirt1) to pharmacologically inhibit the expression of Sirt1. As shown in Figure 7, SAB significantly suppressed the expression of A-p53 compared to the high glucose group, whereas Ex527 reversed the effects of SAB. At the same time, we measured the effects of Ex527 on expression of procaspase-3 and further confirmed that Sirt1 acts on SAB to alleviate the cellular apoptosis induced by HG or PA.

## 4. Discussion

The main purpose of this study was to direct attention to the relationship between Sirt1 and the potential effects of SAB on HUVEC cells treated by high glucose or high fat. We demonstrated that SAB could attenuate endothelial dysfunction by dramatically upregulating expression of Sirt1. Moreover, we found that the inhibitor of Sirt1 abolished protective function of SAB. Our research provided a potential novel approach and mechanism for the treatment of diabetic cardiopathy.

It is well known that endothelial dysfunction is a matter marker of numerous cardiovascular diseases. In an in vitro study, HUVEC cells were cultured under high glucose or high fat conditions to establish a cell model of cardiovascular disease of DM. In previous our study, we demonstrated that salvianolic acid (Sa) could inhibit the vascular endothelial dysfunction induced by high glucose [26]. SAB is one component of Sa; therefore we investigated effect of SAB on diabetic cardiopathy, though high glucose or high fat model to prove that SAB is a major effective component of Sa.

Decreased HUVEC cells proliferation is a major pathological feature in the early stage of cardiovascular disease of DM. The Bcl-2 protein family and caspase family play a key role in the regulation of cell apoptosis [30]. Overexpression of Bcl-2 can inhibit cell apoptosis, and caspases activate the upstream of apoptosis [31]. Moreover, the Bcl-2 control the permeability of mitochondria and the release of cytochrome c to the cytoplasm, following the activation of a group of caspases, which proceeds apoptosis. Among caspases family, caspase-9 and caspase-3 are the most representative [32]. Caspase-3 can activate intrinsic apoptosis, while caspase-9 can increase ROS accumulation. Our data also revealed the potent effect of SAB reversed cell apoptosis induced by high glucose or PA relative to Bcl-2 and caspase families.

As is well known, apoptosis is closely related to oxidative stress. SAB is a new generation of the natural antioxidants, which has a plurality of phenolic hydroxyl group, so it has strong antioxidant activity. It has been reported that SAB inhibits HG-induced oxidative stress and reduces the generation of ROS and mitochondrial depolarization [33]. It well know that ROS of the patients with hyperglycemia or hyperlipidemia is from many different aspects, including protein glycation, PKC activation, the mitochondrial oxidative phosphorylation, and NAD(P)H oxidase activation [34, 35]. Hence, inhibition of ROS overproduction can relief patients with hyperglycemia or depolarization contributed to reduce cellular oxidative stress and promote cells recovery. Thioredoxin-interacting protein (TXNIP), which is an early response gene highly induced by diabetes and hyperglycemia, acts as a mediator of cellular metabolism [36]. TXNIP was initially identified as one of the proteins that interacts with thioredoxin (TRX) and reduces its function which scavenges ROS [37]. It was found that TXNIP mediates glucose-induced apoptotic death in pancreatic beta cells [38]. In our study, we found that SAB could clean away over ROS very well and decreased the expression of TXNIP induced by high glucose or PA.

Sirt1 is a crucial regulator of vascular dysfunction and exerts beneficial effect on glucose-lipid homeostasis in DM. Intriguingly, the inhibition of Sirt1 with pharmacological agent leads to an elevation of ROS levels, indicating a definite relationship between Sirt1 and ROS [39]. The previous experiment proved that Lithospermic acid B can significantly reduce death of beta cells and promote activity of Sirt1 [40]. While SAB also mediates redox state, we deduced that SAB may have a potent ability to upregulate Sirt1 expression. In our experiment, western blot assay showed that expression of Sirt1 was increased by SAB in HUVECs treated with high glucose or PA.

Based on the information stated above, exploring the effect of SAB on Sirt1 expression in HUVEC cells is crucial. To clarify it, we measured expression of Sirt1 in HUVEC cells inhibited by Ex527 or not. The data suggested that inhibition of Sirt1 attenuated SAB function in HUVEC cells treated with high glucose or PA.

## 5. Conclusion

To conclude, the effects of SAB on two HUVEC cells models were investigated in the present study. The results demonstrated that SAB attenuated the HG- or PA-induced cell apoptosis in HUVEC cells via increasing Bcl-2, procaspase-3, and procaspase-9, suppressing TXNIP and activating Sirt1 expressions. The data suggested that SAB might be a promising agent for the therapy of cardiovascular disease of DM. However, the study is a preliminary in vitro study, and further in vivo experiments are needed.

## Data Availability

The data used to support the findings of this study are included within the article.
